# Clinicopathologic features of gastric glomus tumor: A report of 15 cases and literature review

**DOI:** 10.3389/pore.2022.1610824

**Published:** 2023-01-09

**Authors:** Minying Deng, Rongkui Luo, Jie Huang, Yuanlong Luo, Qi Song, Huaiyu Liang, Chen Xu, Wei Yuan, Yingyong Hou

**Affiliations:** ^1^ Department of Pathology, Zhongshan Hospital, Fudan University, Shanghai, China; ^2^ Shanghai University of Traditional Chinese Medicine, Shanghai, China

**Keywords:** glomus tumor, gastric tumor, immunohistochemistry, molecular genetics, prognosis

## Abstract

**Objective:** Glomus tumor is a relatively uncommon soft tissue neoplasm predominantly occurring in upper extremity (fingers), less reported in stomach. This study aimed to discuss the clinicopathologic features of gastric glomus tumor (GGT) and then provide reference for clinical practice.

**Methods:** A retrospective analysis of all cases pathologically diagnosed of GGT was performed, pathological findings were correlated with clinical information, immunohistochemical studies, next-generation sequencing, and patient follow-ups. A review of literature by searching similar cases was conducted to summarize previous knowledge of GGTs.

**Results:** Our study identified 15 GGTs included 5 males and 10 females, aged between 35–75 years old (median, 49 years old). The tumor was located to the gastric corpus in 6 cases (40%) and to the antrum in 9 cases (60%). The maximum tumor diameter ranged between 1–4 cm (median, 1.5 cm). There were 11 cases (73%) of solid glomus tumor, 3 cases (20%) of mixture of solid glomus tumor and glomangioma, and 1 case (7%) of glomangiomyoma. Partial spindle cell area was observed in 3 cases (20%), moderate cellular atypia in 1 case (7%), atypical mitosis in 1 case (7%), vascular invasion in 5 cases (33%), neural invasion in 6 cases (40%) and tumor necrosis in 1 case (7%). Tumor cells expressed Collagen type IV, α-smooth muscle actin (α-SMA), and synaptophysin in most cases. The Ki67 index varied from 1% to 30%. Next-generation sequencing reported *EGFR, PIK3CA, KEAP1* and *TP53* mutation*.* The outcome information was obtained in 12 (80%) cases, followed for 6–63 months, 11 patients (92%) had tumor-free survival and 1 patient (8%) developed liver metastasis 26 months after surgery. Literature review obtained 16 previously reported malignant GGT cases. In terms of the total 31 cases, univariate analysis revealed that the atypical mitosis (OS: *p* = 0.009; DFS: *p* = 0.010) and severe cellular atypia (OS: *p* = 0.007; DFS: *p* = 0.004) were significantly associated with poor prognosis (patient death).

**Conclusion:** GGT is indolent, while long-term close follow-up should be required in the presence of increasing number of risk factors. Malignant GGT is relatively uncommon and predisposes to liver metastasis, calling for accumulation of large-sample data and experience.

## Introduction

Glomus tumor (GT) is a type of mesenchymal neoplasm originating from the glomus cells. It is rare and mainly occurs in the extremities and peripheral soft tissues, rarely in visceral organs. Gastric glomus tumor (GGT) was first reported by Talijeva et al. [1] in 1928. The present study retrospectively analyzed the clinicopathologic characteristics, immunohistochemical features, gene mutation, and prognosis in 15 cases of GGT from Zhongshan hospital. Additionally, literature review was performed to obtain the previous 16 reports of malignant GGT cases, and statistical analysis was conducted, so as to raise the awareness about this disease and provide reference for clinical practice.

## Materials and methods

### Clinical data

Fifteen patients who were histopathologically diagnosed with GGT by the Pathology of Zhongshan Hospital, Fudan University between January 2014 and April 2022 were included. Basic clinical data, including age, sex, tumor site, tumor size, clinical symptom, and previous history, were collected.

### Hematoxylin & Eosin (HE) and immunohistochemical (IHC) staining

HE staining: All specimens were fixed by 10% neutral formalin, regularly dehydrated, paraffin-embedded, sectioned into 3-μm sections, and processed for HE staining. IHC staining: IHC staining was performed using the EnVision two-step strategy. Primary antibody information: CD117, HMB45, Desmin, synaptophysin, chromograninA, S100 protein, wide-spectrum cytokeratin, Ki67, and vimentin (DAKO); Bcl2, P53, CD56, CD57 (LEICA); muscle specific actin (MSA), CD34, H-caldesmon (LongIsland); SOX10, INI1, DOG-1 (Gene Tech); somatostatin receptor 2 (SSTR2), somatostatin receptor 5 (SSTR5) (Abcam); α-smooth muscle actin (α-SMA) (Thermo); ATRX (Sigma); calponin, Collagen type IV (MXB Biotechnologies); BRAF V600E (ZSGB Biotechnology). The dilutions, clone and sources of these antibodies were listed in Table 1.

**TABLE 1:** Primary antibodies used in this study.

Antigen	Clone	Catalogue numbers	Dilution	Source
CD117	Poly	A4502	1: 300	DAKO
HMB45	HMB45	M0634	1: 100	DAKO
Desmin	D33	M0760	1: 100	DAKO
Synaptophysin	DAK-SYNAP	IR660	working fluid	DAKO
S100 protein	Poly	IR504	working fluid	DAKO
wide-spectrum CK	AE1/AE3	M3515	1: 200	DAKO
Ki67	MIB1	RMA-0731	1: 200	DAKO
Vim	V9	M0725	1: 200	DAKO
chromograninA	DAK-A3	M-0202	1: 200	DAKO
Bcl2	3.1	NCL-L-Bcl2	1: 100	LEICA
p53	DO-7	NCL-L-P53-DO7	1: 500	LEICA
CD56	1B6	NCL-CD56-1B6	1: 100	LEICA
CD57	NK-1	NCL-NK1	1: 200	LEICA
MSA	HHF35	M-0002	1: 100	LongIsland
CD34	QBEnd/10	M-0117	1: 100	LongIsland
H-caldesmon	TD107	M-0061	1: 50	LongIsland
SOX10	SDM2	Gt221029	1: 100	Gene Tech
INI-1	25	GT225729	1: 100	Gene Tech
DOG-1	SP31	GT205429	1: 100	Gene Tech
SSTR2	UMB1	ab134152	1: 200	Abcam
SSTR5	UMB4	ab109495	1: 200	Abcam
SMA	1A4	MS-113-P	1: 100	Thermo
ATRX	Poly	HPA001906	1: 500	SIGMA
Calponin	CALP	GM355629	1: 200	MXB Biotechnologies
Collagen type	IV	MAB0781	working fluid	MXB Biotechnologies
BRAF V600E	RMD15	ZM-0632	working fluid	ZSGB Biotechnology

### Next-generation sequencing

DNA was extracted from the paraffin-embedded tissue samples of 15 GGT, and then processed for analysis on *BRAF* gene using the PCR-Sanger sequencing and amplification refractory mutation system (ARMS) using the ABI3500Dx sequencing platform and ABI7500 sequencing platform, respectively. At the same time, the Illumina Nextseq CN500 detection platform was used to amplify 15 cases of GGT (Amplicon-based NGS).

### Histological assessment

The HE-stained sections were assessed from the following aspects: 1) histological type; 2) growth pattern (infiltrative/expansive): infiltrative growth was defined by unclear borders between tumor tissues and surrounding normal tissues, and the growth of tumor tissues between intermuscular or interfascial spaces; expansive growth was defined by clear borders between tumor tissues and surrounding normal tissues; 3) infiltration level: muscular infiltration was defined by tumor cells growing to smooth muscle fibers in a nested, lingual, or patchy manner; 4) myxoid degeneration of the stroma; 5) spindle cell area; 6) cellular atypia: the degree of cellular (nuclear) atypia was determined by previous experience and the study of Folpe et al. [2]. Mild atypia: tumor cells are similar to the normal glomus cells, presenting with a small regular nucleus with an unobvious nucleolus, or a transverse diameter of the tumor cell nucleus ≤ diameter of 1 lymphocyte; moderate atypia: occasional nucleolus, or diameter of 1 lymphocyte < transverse diameter of the tumor cell nucleus ≤ diameter of 2 lymphocytes; severe atypia: the tumor cell nucleus is 2–3 times larger than the normal glomus cells and is significantly irregular with an obvious nucleolus, or transverse diameter of the tumor cell nucleus > diameter of 2 lymphocytes [2, 3]; 7) mitotic counts from 50 high power fields (HPFs), magnification of 400 (OLYMPUS BX43, FN = 22 or LEICA DM2000, FN = 22) (50HPFs = 12 mm^2^); 8) atypical mitosis; 9) vascular invasion; 10) neural invasion; 11) tumor necrosis; 12) mucosal erosion or ulceration; 13) hemorrhage; 14) calcification.

### Literature retrieval

The second part of this study was made up of 16 malignant GGT cases between 2001–2022 retrieved from the PubMed and Wanfang databases from different hospitals and institutes. The same basic and clinicopathologic data of all retrieved cases as those analysed in our series of cases were collected by going through the original reference [2–16].

### Follow-up

Data about follow-up were obtained by review of medical records and telephone interviews. Patients were followed up in months from the day after pathological confirmation to 27 May 2022. Termination of the follow-up was determined by loss-to-follow-up or death. Primary endpoints were overall survival (OS) and disease-free survival (DFS). DFS is defined by the time period from the date of surgery to the date of the first tumor metastasis/recurrence or the last follow-up visit. OS is defined by the time period from the date of surgery to the date of death from tumor or the last follow-up visit.

### Statistical analysis

IBM SPSS Statistics 25 (IBM, China) software was used for statistical analysis. Pearson correlation analysis was applied to assess the correlation between continuous data (including age, maximum tumor diameter, and mitotic count). Other clinicopathologic data were analyzed by Crosstabs and Fisher exact probability test, and non-parametric Spearman test was used for correlation analysis. Prognostic significance of the clinicopathologic parameters for the clinical outcomes of the disease (recurrence/metastasis, death) was assessed by Kaplan-Meier survival curve together with Log-rank test. Multivariate analysis was performed using the Cox proportional hazard regression analysis. *p* < 0.05 was considered as statistically significant. *p*-value for trend was between 0.05–0.1. The range of r value for the correlation coefficient is between −1 and 1, and the larger the absolute value, the stronger the correlation.

## Results

### Clinical characteristics

Of the 15 GGT patients from Zhongshan hospital, there were 5 males (33%) and 10 females (67%). The age of onset was between 35–75 years old (median, 49 years; average, 51.6 years). Tumor sites were the gastric corpus (*n* = 6, 40%) and the antrum (*n* = 9, 60%). One patient (7%) was admitted for repeated hematemesis with black stool for half a month, 1 (7%) for progressive elevation of carcinoembryonic antigen (CEA) for 5 years (Pre-operative >9 ng/mL, post-operative 7–8 ng/mL), 4 (27%) for recurrent upper abdominal discomfort, and 9 (60%) for a stomach-occupying lesion found on physical examination. Previous histories included alcohol consumption (*n* = 3, 20%), smoking (*n* = 2, 13%), hypertension (*n* = 1, 7%), diabetes (*n* = 1, 7%), malignant thyroid tumor (*n* = 1, 7%), meningioma (*n* = 1, 7%), and duodenal gastrointestinal stromal tumor (GIST) with hypertension, hypokalaemia and thymoma, without any GIST familiar predisposition, Carney syndrome or anamnestic data regarding genetic background (*n* = 1, 7%) (Table 2).

**TABLE 2:** Clinical symptom and surgical procedures of the 15 GGTs from Zhongshan hospital.

Case	Sex	Age(y)	Clinical symptom	Preoperative diagnosis	Surgical procedure
1	F	59	A 10-year stomach-occupying lesion found on physical examination	GIST	Subtotal gastrectomy
2	F	49	Half-month recurrent hematemesis with black stool	Gastric malignancy with hemorrhage	Radical gastrectomy
3	F	57	Over 4 years of recurrent upper abdominal discomfort	A submucosal benign mass of the antrum	EFTR
4	M	65	An 8-month gastric submucosal mass found on physical examination	GIST	EFTR
5	M	49	A 10-year stomach-occupying lesion found on physical examination	GIST	Laparoscopic gastrectomy
6	F	35	2-year gastric submucosal protrusion on physical examination	GIST	ESD
7	M	50	5-year progressive CEA elevation on physical examination	Neuroendocrine neoplasm	ESD + subtotal gastrectomy + sentinel lymph-node dissection
8	M	62	A 2-month gastric mass on physical examination	Lipoma	EFTR
9	F	75	A stomach-occupying lesion found on physical examination	GIST	EFTR
10	M	58	Intermittent abdominal pain with no predisposing factors, accompanying postprandial acid regurgitation for over half a year	Neuroendocrine neoplasm	Subtotal antrectomy + subtotal duodenostomy
11	F	41	Left upper abdominal pain for over 3 years and exacerbation for 4 years	GIST	Laparoscopic gastrectomy
12	F	41	A 4-month submucosal mass localized to the posterior wall of the antrum found on physical examination	GIST	ESD
13	F	51	3-year submucosal protrusion of the antrum on physical examination	GIST, hemangioma	ESD
14	F	36	A stomach-occupying lesion found on physical examination	SMT of the gastric corpus	Subtotal gastrectomy
15	F	46	Upper abdominal pain for over 2 months	SMT of the gastric corpus	ESD

### Imaging and surgical procedures

**FIGURE 1:**
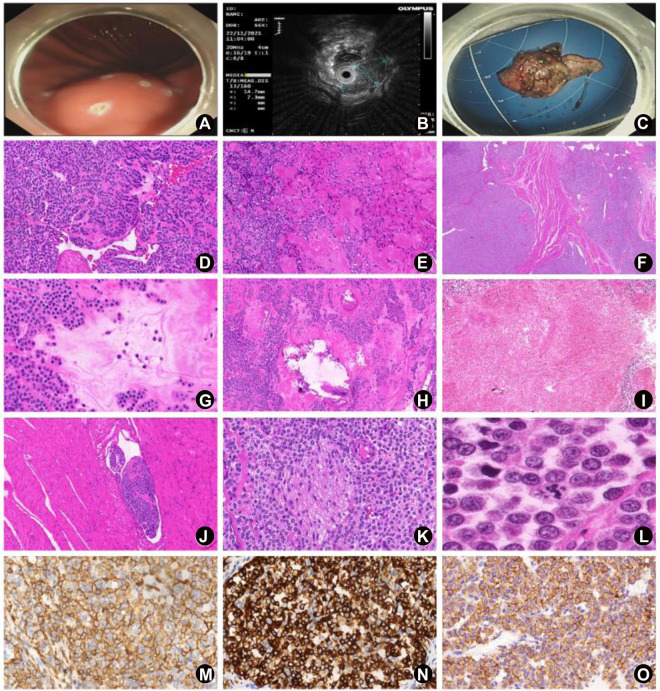
Clinical features, HE, and IHC results of the 15 GGT cases. **(A)** Case 15: a 2-cm submucosal protrusion localized to the posterior wall of the upper part of the gastric corpus on gastroscopic examination, with a smooth surface and a soft texture. **(B)** Case 15: a 15 ⅹ 8 mm uniformed hypoechoic occupying lesion on ultrasound gastroscopy, with clear borders, originating from the submucosal layer with intact posterior mucosal layer. **(C)** Case 15: endoscopic full-thickness resection (EFTR) of the mass, with absence of partial muscular layer. **(D)** Case 10: solid glomus tumor growing surrounding the blood vessels in a patchy consolidation pattern (HE, high magnification). **(E)** Case 4: glomangiomyoma showing a transition state of glomus cells and smooth muscle cells (HE, high magnification). **(F)** Case 1: solid glomus tumor invading to the muscular layer (HE, medium magnification). **(G)** Case 10: myxoid degeneration of the partial stroma (HE, high magnification). **(H)** Case 10: calcification in part of the tumor (HE, high magnification). **(I)** Case 14: necrosis in part of the tumor (HE, medium magnification). **(J)** Case 10: vascular invasion (HE, medium magnification). **(K)** Case 1: neural invasion (HE, high magnification). **(L)** Case 14: visible atypical mitosis (HE, high magnification); **(M)** Diffuse expression of α-SMA by tumor cells (EnVision, high magnification). **(N)** Diffuse expression of synaptophysin by tumor cells (EnVision, high magnification). **(O)** Diffuse expression of SSTR2 by tumor cells (EnVision, high magnification).

Nine patients (60%) underwent preoperative CT, and 8 of them manifested spindle or circle soft-tissue density shadow in the gastric wall, which was significantly enhanced after enhanced scan (Figure 1A). Six patients (40%) were examined by endoscopic ultrasonography preoperatively, showing a gastric wall submucosal tumor (SMT) originating from the submucosal layer or the muscularis propria (Figure 1B). Eleven patients (73%) received preoperative endoscopy and presented with mucosal protrusion in the gastric wall, which had a smooth surface and was complicated by partial hemorrhage. Combined with imaging and clinical presentations, 8 patients (53%) were diagnosed with GIST, 2 (13%) with neuroendocrine tumor, and 1 (7%) with lipoma before surgery. Additionally, 1 patient (7%) was considered as having gastric malignancy, while the other 3 patients (20%) were considered as having gastric SMT on imaging before surgery. Surgical procedures were endoscopic full-thickness resection (EFTR) under general anesthesia in 4 patients (27%), endoscopic submucosal dissection (ESD) in 4 patients (27%), subtotal gastrectomy in 2 patients (13%), radical gastrectomy in 1 patient (7%), laparoscopic gastric tumor resection in 2 patients (13%), subtotal antrectomy plus subtotal duodenostomy for concurrent occupying lesions in the stomach (GGT) and duodenum (GIST) in 1 patient (7%), and tumor resection followed by subtotal gastrectomy plus sentinel lymph-node dissection in 1 patient (7%) (neuroendocrine tumor was considered by biopsy pathology, and the resection margin was positive) (Figure 1C) (Table 2).

### Pathologic features

#### Gross examination

Tumors of 14 patients (93%) were single and nodular, presenting with greyish white and yellow sections with partially colored greyish red, a soft and tough texture, and clear borders. The tumor of the other 1 patient (7%) was polypoid colored grayish brown, without a well-defined base. None of the tumors had erosion or ulceration. The maximum tumor diameter ranged between 1–4 cm (median, 1.5 cm; average, 1.89 cm), less than 2 cm in 9 patients (60%) while between 2–5 cm in 6 patients (40%).

#### Microscopic examination

At low magnification, gastric mucosal tissues were absent in 6 cases (40%), possibly because it was not removed during the surgical section or taking the pathological specimen. Gastric mucosal tissues were observed in 9 cases (60%), but none of them had mucosal erosions. The muscularis propria layer and the subserosal layer of the gastric wall were involved in 12 cases (80%) and 2 cases (13%), respectively. It was impossible to judge on the involvement in the other 1 case (7%) because of the fragmented specimen without proper histological orientation. Tumors in 6 cases (40%) presented with an expansive growth pattern, and tumors in 8 cases (53%) showed an infiltrative growth pattern, including the tumor in 1 case exhibiting patchy consolidation (40%) for not abundant blood sinus inside the tumor (Figure 1F). The other 1 case (7%) was unable to be assessed due to the crushed tumor tissues and absence of normal tissues surrounding the tumor. There were 11 cases (73%) of typical solid glomus tumor, 3 cases (20%) of mixture of solid glomus tumor and glomangioma, and 1 case (7%) of glomangiomyoma (Figures 1D,E). Hemorrhage was seen from the inner area or margin of the tumor in 3 cases (20%), and small focal calcification was observed in the inner part of the tumor in 3 cases (20%) (Figure 1H). Hyalinosis or myxoid degeneration of the stroma (2%–30%) were detected in 13 cases (87%), and spindle cell areas in partial parts of the tumor (2%–5%) were noticed in 3 cases (20%) (Figure 1G). At high magnification, 14 cases (93%) exhibited mild nuclear atypia, and the other one case (7%) showed moderate atypia. Mitosis was readily visible in 5 cases (33%) (1-6 mitoses/50 HPFs or 12 mm^2^), including atypical mitosis in only 1 case (Figure 1L). Vascular invasion and neural invasion were observed in 5 cases (33%) and 6 cases (40%), respectively (Figures 1J,K). Only one case (7%) presented with focal tumor necrosis (about 10%) (Figure 1I). Ten cases (67%) showed visible neuroendocrine tumor-like cells surrounding the tumor (2%–20%), which had scanty cytoplasm, hyperchromatic nuclei and fine chromatin. Three cases (20%) had tumor tissues in the resected margin (Table 3).

**TABLE 3:** Clinicopathologic features of the 15 GGTs from Zhongshan Hospital.

Case	Gastric site	Size (cm)	Location	Infiltrative growth	Mitoses/50HPF	Risk indicator							Diffuse growth inside the tumor	Myxoid degeneration of the stroma	Mucosal erosion or ulceration	Histological subtype	Follow-up
						Severe atypia	Mitoses>5/50 HPF	Atypical mitoses	Vascular invasion	Neural invasion	Tumor necrosis	Cumulative number					
1	Corpus	1.5	Muscularis propria	No	0	No	No	No	No	Yes	No	1	No	Yes, 5%	Yes	solid glomus tumor	Loss-to- follow-up
2	Antrum	2.7	Muscularis propria	Yes	0	No	No	No	Yes	No	No	1	No	Yes, 30%	No	solid glomus tumor	ANED, 63 months
3	Antrum	2.5	Muscularis propria	Yes	0	No	No	No	No	No	No	0	No	Yes, 20%	No	solid glomus tumor	ANED, 59 months
4	Antrum	1.2	Muscularis propria	Yes	0	No	No	No	No	No	No	0	No	Yes, 30%	No mucosa	Glomangiomyoma	ANED, 61 months
5	Corpus	2.3	Subserosa	Yes	1	No	No	No	Yes	No	No	1	No	No	No	solid glomus tumor	Loss-to-follow-up
6	Antrum	1	—	—	0	No	No	No	No	No	No	0	No	Yes, 10%	No	solid glomus tumor + glomangioma	ANED, 57 months
7	Corpus	1	Muscularis propria	No	0	No	No	No	No	Yes	No	1	No	Yes, 20%	No mucosa	solid glomus tumor + glomangioma	ANED, 46 months
8	Antrum	1.2	Muscularis propria	No	0	No	No	No	No	Yes	No	1	No	Yes, 2%	No	solid glomus tumor	ANED, 37 months
9	Corpus	4	Muscularis propria	No	0	No	No	No	No	No	No	0	No	Yes, 5%	No	solid glomus tumor	ANED, 26 months
10	Antrum	1	Muscularis propria	Yes	0	No	No	No	Yes	Yes	No	2	No	Yes, 10%	No mucosa	solid glomus tumor	ANED, 27 months
11	Antrum	2	Muscularis propria	Yes	0	No	No	No	No	Yes	No	1	No	Yes, 2%	No mucosa	solid glomus tumor+ glomangioma	Loss-to-follow-up
12	Antrum	1.5	Muscularis propria	No	2	No	No	No	Yes	No	No	1	No	Yes, 2%	No mucosa	solid glomus tumor	ANED, 16 months
13	Antrum	1	Muscularis propria	Yes	1	No	No	No	No	Yes	No	1	No	Yes, 5%	No mucosa	solid glomus tumor	ANED, 9 months
14	Corpus	4	Subserosa	Yes	6	No(moderate)	Yes	Yes	Yes	No	Yes, 10%	4	Yes, 40%	Yes, 5%	No	solid glomus tumor	ANED, 31 months; liver metastasis, 26 months postoperatively
15	Corpus	1.5	Muscularis propria	No	2	No	No	No	No	No	No	0	No	Yes, 2%	No	solid glomus tumor	ANED, 6 months

Note: —: Non-evaluable due to the fragmented tissues and absence of normal tissue surrounding the tumor; ANED: alive with no evidence of disease; No mucosa: Not seen under the microscope and not clear whether caused by ulceration.

### Immunophenotype

The immunohistochemical results are summarized in Table 4 and Figures 1M–O. Vimentin was expressed by all the 15 cases (100%); α-SMA and SSTR2 were expressed by 14 cases (93%). Variable Collagen type IV positivity (median percentage of positive cells 90%) was seen in all 15 cases. Positive expression of H-caldesmon and synaptophysin were detected in 13 cases (87%), of which synaptophysin positivity varied from 10% of tumor cells to global immunoreactivity (median of positive tumor cells 70%). CD57 and MSA was expressed in 12 cases (80%); ten of 15 tumors (67%) had different positivity for Bcl2 in 2%–100% of tumor cells. Calponin were expressed in 9 cases (60%) (median 20% of positive cells); partial or focal expression of DOG-1 was observed in 5 cases (33%), varying from 5% of tumor cells to 20%, whereas of those cases CD117 negative and Collagen type IV positive, so it could be differentiated from GIST. Focal expression of CD34 and CD56 was noted in 2 cases (13%), of which Collagen type IV was positive in CD34-positive partly cases, so it could be differentiated from solitary fibrous tumor. S-100 protein, desmin, chromograninA, CD117 and HMB45 were slightly expressed by 1 case (7%). Wide-spectrum cytokeratin, SSTR5, Braf (V600E) and SOX10 were negative in all the 15 cases (100%). The Ki67 index ranged between 1%–30%.

**TABLE 4:** Immunohistochemical and molecular results of 15 GGTs from Zhongshan hospital.

Case	1	2	3	4	5	6	7	8	9	10	11	12	13	14	15	Positive/total (% of positive cases)
Molecular genetics	EGFR	−	−	PIK3CA, MSI	−	−	TP53	−	−	−	−	−	−	KEAP1	−	
α-SMA	+/−	70%+	70%+	80%+	100%+	80%++	70%+	30%+	90%+	60%+	40%+	10%+	80%+	60%+	70%+	14/15 (93)
MSA	10%+	90%+	80%+	−	90%++	60%+	−	50%+	80%+	70%+	−	60%+	50%+	30%+	80%+	12/15 (80)
Vimentin	90%++	90%+	70%+	70%+	100%++	80%+	70%+	90%+	70%+	80%+	80%+	20%+	70%+	80%+	30%+	15/15 (100)
H-caldesmon	80%+	30%+	30%+	70%++	20%+	−	−	40%+	30%+	30%++	20%+	30%+	60%+	5%+	20%+	13/15 (87)
calponin	30%+	−	20%+	−	10%+	70%+	−	30%+	−	10%+	−	10%+	10%+	−	30%+	9/15 (60)
Collagen type IV	40%+	100%+	100%++	80%+	60%+	70%+	40%+	90%+−++	100%+	100%++	100%++	80%+	100%+	100%++	90%++	15/15 (100)
CD34	−	15%+	−	−	−	−	−	−	−	−	−	−	20%+	−	−	2/15 (13)
Desmin	−	−	−	−	−	−	−	−	−	−	−	−	−	−	5%+	1/15 (7)
S100 protein	−	−	−	−	−	20%+	−	−	−	−	−	−	−	−	−	1/15 (7)
wide-spectrum cytokeratin	−	−	−	−	−	−	−	−	−	−	−	−	−	−	−	0/15 (0)
Synaptophysin	100%++	−	60%+	10%+	80%+	70%+	80%+	60%+	80%+	80%+	40%+	50%+	−	90%+	70%+	13/15 (87)
CgA	−	−	−	−	−	−	−	−	−	−	−	−	20%+	−	−	1/15 (7)
CD56	−	−	−	−	−	−	−	−	−	−	−	10%+	−	−	5%+	2/15 (13)
Bcl2	−	−	−	5%+	100%++	70%+	50%+	−	40%+	−	30%+	2%+	60%+	80%+	70%+	10/15 (67)
P53	2%+	20%+	60%+	10%+	40%+	60%+	−	5%+	20%+	5%+	5%+	10%+	30%+	−	70%+	
Braf(V600E)	−	−	−	−	−	−	−	−	−	−	−	−	−	−	−	0/15 (0)
INI1	90%+	70%+	90%+	80%+	90%+	80%+	80%+	50%+	80%+	90%+	90%+	90%+	90%+	90%+	100%+	15/15 (100)
CD57	−	20%+	70%+	80%+	−	70%+	40%+	80%+	70%+	80%+	80%+	10%+	60%+	100%+	30%+	12/15 (80)
Ki67	1%	5%	Dense area 10%	1%	5%	2%	3%	5%	5%	5%	3%	5%	2%	10%	5%	
SSTR2	80%+	70%+	70%+	50%++	10%+	60%+	−	80%+	95%++	80%+	70%+	100%++	95%++	10%+	90%+	14/15 (93)
SSTR5	−	−	−	−	−	−	−	−	−	−	−	−	−	−	−	0/15 (0)
CD117	−	−	−	−	−	−	10%+	−	−	−	−	−	−	−	−	1/15 (7)
DOG-1	−	−	−	−	−	−	−	10%+	−	10%+	5%+	20%+	−	−	10%+	5/15 (33)
SOX10	−	−	−	−	−	−	−	−	−	−	−	−	−	−	−	0/15 (0)
HMB45	−	−	−	10%+	−	−	−	−	−	−	−	−	−	−	−	1/15 (7)

### Molecular genetics

Sanger sequencing and ARMS testing demonstrated no *BRAF V600E* mutation in all 15 cases (100%). High-throughput sequencing technology found one case of *EGFR* gene exon 21 R831H missense mutation. One case detected the *PIK3CA* gene exon 10 E545K missense mutation, and found microsatellite instability (MSI). One case found a *TP53* gene exon 5 T150Afs*16 frameshift mutation. One case found that exon 3 of the *KEAP1* gene P384 = synonymous mutation (Table 4).

### Follow-up and prognosis

None of the 15 patients from Zhongshan hospital underwent postoperative radiotherapy or chemotherapy. Twelve patients (80%) completed follow-up by telephone interviews or outpatient visit, and the follow-up time period was between 6–63 months. Of the 12 patients, 1 patient underwent hepatectomy for localized liver metastases 26 months postoperatively and survived after 31 months. The other 11 patients had tumor-free survival. Two of them underwent ESD and one was treated by EFTR under general anesthesia. All the three patients had visible tumor tissues in the resected margin endoscopically, and survived 57, 37, and 9 months postoperatively without recurrence or metastasis (Table 3).

### Clinical pathological and follow-up data of 16 cases of malignant GGT reported in the literature

Literature review of the current study obtained 16 cases of malignant GGT reported domestically and abroad between 2001 and 2022, together with their clinicopathologic and follow-up data [2–16]. Among the 16 patients, there were 7 males (44%) and 9 females (56%) (male-to-female ratio = 1:1.3), aged between 18–80 years old (median, 63 years). The tumors were localized to the gastric antrum (*n* = 7, 44%), the corpus (*n* = 3, 19%), and the fundus (*n* = 3, 19%). No specific tumor site was reported in the other 3 cases (19%). The maximum tumor diameter ranged between 2–17 cm (median, 5.65 cm). Involved sites included the muscularis propria (*n* = 4, 25%), the serosal layer (*n* = 2, 13%), the mucosal muscle layer (*n* = 1, 6%), and the submucosal layer (*n* = 1, 6%) of the gastric wall. No specific involvement was mentioned in the reports of the other 8 cases (50%). Mucosal erosion or ulceration was visible in 5 cases (31%), myxoid degeneration of the stroma in 2 cases (13%), partial spindle cell areas in 6 cases (38%), partial tumor necrosis in 2 cases (15%), and vascular invasion in 6 cases (38%). None of the 16 cases reported neural invasion. In terms of the cellular atypia, it was presented as severe in 8 cases (50%), moderate in 4 cases (25%), and mild in 2 cases (13%). In the other two cases, cellular atypia was not available from primary literature. 13 cases (81%) showed mitosis, of which 9 cases used 50HPF count with the range of 1-15/50HPF, and 4 cases used 10HPF count with the range of 9-28/10HPF. Mitosis was not easy to find in one case (6%) and was not mentioned in the other 2 cases (13%) in the original data of literature. Ki67 cell proliferation index was reported in 5 cases, ranging from 10% to 40%. Follow-up was completed in 14 cases (88%), ranging between 0.2–72 months (median, 9.5 months; average, 21.7 months). Nine patients (56%) experienced metastasis, majorly to the liver (the most common), lung, brain, kidney, scalp, humerus, epididymis, and greater omentum. One patient developed liver metastasis 36 months postoperatively and eventually died. Another one patient suffered from multiple metastases to the liver, lung, brain, and scalp, and eventually died as well (Table 5).

**TABLE 5:** Clinicopathologic features of the 16 previously reported cases of malignant GGT from different institutes.

Case	Author	Publication year	Sex	Age(y)	Tumor site	Diameter (cm)	Location	Nuclear grade	Risk indicator							Spindle cell area	Myxoid degeneration of the stroma	Ki67-index	Follow-up
									Severe atypia	Mitoses/10 or 50 HPF	Atypical mitoses	Vascular invasion	Neural invasion	Tumor necrosis	Cumulative number				
1	Folpe[2]	2001	M	69	Stomach	8.5	NA	Mild	No	3/50	No	Yes	NA	No	1	No	NA	NA	DOD, 36 months; liver metastasis in the same month
2	Miettinen[3]	2002	M	69	Antrum	6.5	Serosa	Mild	No	1/50	NA	Yes	NA	NA	1	Yes	NA	2%	DOD, 50 months; liver metastasis, 33 months postoperatively
3	Bray[4]	2009	M	58	Stomach	17	Serosa	Moderate	No	>15/50	NA	Yes	NA	NA	2	NA	NA	NA	DOD, 72 months; liver, lung, brain, scalp metastases in the same month
4	Lee[5]	2009	F	65	Gastric fundus	3	NA	High	Yes	2/50	Yes	NA	NA	NA	2	NA	NA	NA	DOD, 8 months; kidney and brain metastases at the same time, bone metastasis 4 months postoperatively
5	Lee[5]	2009	M	63	Gastric corpus	9	NA	High	Yes	NA	NA	Yes	NA	NA	2	NA	NA	NA	DOD, 2 months; liver metastasis at the same time
6	Zhang[6]	2009	F	18	Greater curvature in the posterior wall	5	NA	High	NA	2–3/50	NA	NA	NA	NA	0	Yes	Yes	NA	Survival 72 months postoperatively; metastasis to left upper uterus and greater omentum in the same month
7	Song[7]	2010	F	65	Gastric fundus	3	Muscularis propria	Moderate	Yes	2/50	Yes	NA	NA	NA	2	Yes	NA	NA	DOD, 7 months; bone, epididymis, and bilateral lung metastases 1 month postoperatively; brain metastasis 6 months postoperatively
8	Teng[8]	2012	F	66	Antrum	5.3	Muscularis propria	High	No	4/50	NA	Yes	NA	NA	1	Yes	NA	NA	ANED, 9 months
9	Yao[9]	2013	F	52	Antrum	2	NA	High	Yes	0	NA	NA	NA	NA	1	NA	Yes	NA	ANED, 13 months
10	Tao[10]	2013	F	36	Antrum	4	Muscularis propria	High	NA	<5/50	NA	NA	NA	NA	0	NA	NA	NA	ANED, 10 months
11	Zaidi[11]	2016	F	53	Gastric fundus	10	Submucosa	High	Yes	10/50	Yes	NA	NA	Yes	4	NA	NA	15%	ANED, 15 months
12	Li[12]	2017	M	53	Antrum	3	Muscularis propria	NA	Yes	NA	NA	Yes	NA	NA	2	NA	NA	10%	NA
13	Bodolan[13]	2018	F	80	Antrum	7.1	NA	High	No	28/10	NA	No	NA	No	1	NA	NA	NA	ANED, 6 days; introoperative liver metastasis
14	Toti[14]	2019	M	72	Gastric corpus	6	NA	Moderate	Yes	14/10	NA	NA	NA	Yes	3	Yes	NA	25%	Survival 3 months postoperatively; liver metastasis before diagnosis of the primary tumor
15	Kong[15]	2019	F	63	Antrum	2.4	Muscularis mucosa	Moderate	Yes	9/10	NA	No	No	NA	2	NA	NA	40%	NA
16	Alsahwan[16]	2021	M	56	Stomach	7	NA	NA	No	12/10	Yes	NA	NA	NA	2	Yes	NA	30%	ANED, 6 months

Note: M, male; F, female; NA, not available; ANED, alive with no evidence of disease; DOD, died of diseases.

### Correlation between clinicopathologic parameters

The total 31 cases, including 15 cases analyzed in the present study from Zhongshan hospital and 16 previously reported cases from literature review between 2001–2022, were analyzed collectively from the following aspects: age, sex, maximum tumor diameter, involvement level, growth pattern, patchy consolidation, cellular atypia, mitotic count, atypical mitosis, vascular invasion, neural invasion, tumor necrosis, spindle cell area, and myxoid degeneration of the stroma. Statistically, significant positive associations were demonstrated between maximum tumor diameter and cellular atypia (*p* = 0.002, r = 0.546), maximum tumor diameter ≥5 cm and spindle cell area (*p* = 0.023, r = 0.528), severe atypia and atypical mitosis (*p* = 0.009, r = 0.728), severe atypia and tumor necrosis (*p* = 0.018, r = 0.792), atypical mitosis and spindle cell area (*p* = 0.012, r = 0.721), tumor necrosis and spindle cell area (*p* = 0.044, r = 0.658). Additionally, remarkable negative associations were indicated between subserosal involvement and myxoid degeneration of the stroma (*p* = 0.011, r = −1.00), and between muscular involvement and severe cellular atypia (*p* = 0.029, r = −0.669).

### Correlation between clinicopathologic parameters and clinical outcome

**FIGURE 2:**
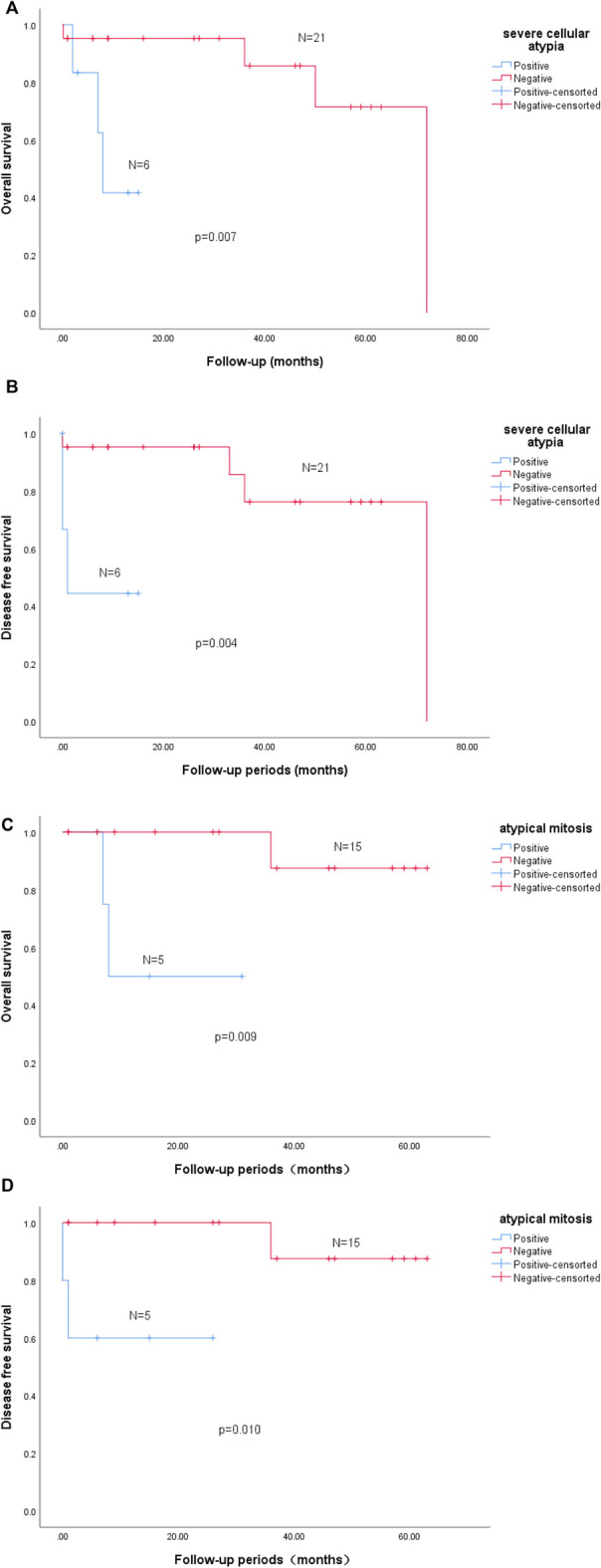
Survival analysis. Kaplan-Meier estimation of overall survival (OS) by severe cellular atypia in GGT (*n* = 27, **(A,B)**). Severe cellular atypia positive group exhibited a shorter DFS (*p* = 0.004, log-rank, *n* = 27, **(B)**) and a significant tendency towards a poor OS (*p* = 0.007, log-rank, *n* = 27, **(A)**). Kaplan-Meier estimation of overall survival (OS) by atypical mitosis in GGT (*n* = 20, **(C,D)**). Atypical mitosis positive group exhibited a shorter DFS (*p* = 0.010, log-rank, *n* = 20, **(D)**) and a significant tendency towards a poor OS (*p* = 0.009, log-rank, *n* = 20, **(C)**).

Associations between the clinicopathologic parameters and clinical outcomes of patients were analyzed in the total 31 cases (Table 6). The results demonstrated that patients with severe cellular atypia predicted shorter OS (*p* = 0.007) and DFS (*p* = 0.004) than patients without severe cellular atypia (Figures 2A,B). In addition, atypical mitosis tended to have shorter OS (*p* = 0.009) and DFS (*p* = 0.010) (Figures 2C,D). Univariate analysis indicated that maximum tumor diameter ≥5 cm was significantly positively associated with the death outcome in patients (*p* = 0.030, r = 0.438). While in multivariate analysis, no statistical significance was found in associations between the adverse clinical outcomes of the disease and the clinicopathologic parameters.

**TABLE 6:** Kaplan-Meier analysis of DFS and OS in total 31 GGT cases.

Parameter	*p*-value (DFS)	*p*-value (OS)
Atypical mitosis	0.010	0.009
Vascular invasion	0.115	0.175
Tumor necrosis	0.665	0.665
Spindle cell area	0.289	0.534
Gender	0.454	0.388
Severe cellular atypia	0.004	0.007
Maximum tumor diameter ≥5 cm	0.073	0.090
Myxoid degeneration of the stroma	—	—
Neural invasion	—	—
Muscularis propria layer involvement	0.819	0.803
Subserosal layer involvement	0.351	0.495
Growth pattern (infiltrative/expansive)	—	—

Note: —: not be statistically analyzed with the available data.

## Discussion

GT is derived from glomus cells that is rare and comprises less than 2% of all soft tissue tumors [17]. It was first reported by Masson et al. [18] in 1924. Together with cutaneous leiomyoma, angiolipoma, and traumatic neuroma, it is also known as “painful subcutaneous nodule.” GT commonly occurs in the extremities and peripheral soft tissues, such as the glomus cell-rich parts including figure, toe, and nail bed, rarely seen in the visceral regions that have fewer glomus cells (approximately 1/4) [19].

GGT is extremely rare accounting for approximately 2.2% of the total gastric tumors while 1% of the gastric mesenchymal tumors [20, 21]. It was first reported by Talijeva et al. [1] in 1928, followed by Kay et al. [22] in 1951, who reported 3 GGT cases. According to the literature, GGT is prevalent in females, with the age of onset ranging between 19–90 years old (median, 55 years; average, 53 years) and the common site being the gastric antrum [3]. The majority of GGT cases have non-specific clinical symptoms, mainly presenting with varying degrees of upper abdominal discomfort, abdominal distension, gastrointestinal bleeding, black stool, and vomiting. Additionally, GGT cases can also be asymptomatic and be found incidentally on physical examination. In the 15 GGT patients reported here from Zhongshan hospital, the male-to-female ratio was 1:2, the age of onset was between 35–75 years old (median, 49 years), and the tumor site was more common in the gastric antrum (*n* = 9), consistent with the literature above. With reference to the cause of admission, 5 patients were admitted for gastrointestinal symptoms, 1 patient for progressive CEA elevation, and 9 patients for a stomach-occupying lesion incidentally found on physical examination, which might be attributed to the popularization of endoscopic examination and the increasing health awareness in people. Usually, GGT patients more often present with mucosal protrusion on gastroscopic examination; a submucosal or muscular uniform hypoechoic mass on ultrasound gastroscopic examination; low-density shadow on CT scan, with clear borders and regular shape, and arterial-phase hyperenhancement after enhanced scan. The imaging presentations of the 15 cases reported here are in line with the literature report. Furthermore, 8 patients (53%) were preoperatively diagnosed with GIST, 2 (13%) with neuroendocrine tumor, and 1 (7%) with lipoma. Additionally, 1 patient (7%) was considered as having gastric malignancy, while the other 3 patients (20%) were considered as having SMT. As GGT is relatively rare, we believed that the clinical symptoms and preoperative imaging presentations mentioned above are not sufficient to make a definite diagnosis of GGT. Instead, histopathology is required. However, GGT should also be well distinguished from the neuroendocrine tumor, GIST, solitary fibrous tumor, paraganglioma, epidermal leiomyoma, or clear cell leiomyoma in their pathology patterns.

Literature has reported that the maximum tumor diameter of GGT is between 2–5 cm (median, 2–3 cm). In most cases, the GGT tumors are solitary with clear borders, greyish red section, and a slightly tough texture, accompanying partial hemorrhage or calcification [3, 23]. In the present study, the maximum tumor diameter of the 15 cases ranged between 1–4 cm (median, 1.5 cm). GGT shares a similar histological pattern with other GTs originating from soft tissues and can be classified as solid glomus tumor, glomangioma, and glomangiomyoma. Here, there were 11 (73%) cases of solid glomus tumor, 3 (20%) cases of mixture of solid glomus tumor and glomangioma, and 1 (7%) case of glomangiomyoma. Hyalinosis or myxoid degeneration of the stroma (2%–30%) were detected in 13 cases (87%), and calcification was observed in 3 cases (20%). By now, there has been no literature reporting the practical significance of the histological subtype and myxoid degeneration of the stroma in clinical prognosis of GGT.

The majority of GGT cases are reported as benign, and malignant GGT is relatively rare. For the first time, Kirschbaum et al. [24] reported a case of malignant GGT in 1939. Subsequently, Yannopoulos et al. [25] reported a 12-year-old girl suffering from malignant GGT in 1962. It has not been established about the standard for definite diagnosis of malignant GGT because of the rare cases. In 2001, Folpe et al. [2] retrospectively analyzed the clinicopathologic parameters of 52 malignant GT cases, including tumor size, infiltration depth, growth pattern, cellular atypia, mitotic count/50 HPFs, atypical mitosis, vascular invasion, and tumor necrosis. Of the 52 patients, 35 patients were followed up for 5 months to 23 years (average, 5.5 years), including 7 patients having recurrence, 8 patients with metastasis, and 7 deaths. Following statistical analysis, they proposed a standard for the diagnosis of malignant GT, including the following three criteria: 1) the tumor localized to the fascia or viscera, with the maximum diameter >2 cm; or 2) visible atypical mitosis; or 3) moderate-to-severe nuclear atypia, and mitotic count ≥5/50 HPFs. The authors noted that patients could be diagnosed with atypical GT if they met one of the following criteria: 1) a deep tumor site; or 2) diameter >2 cm; or 3) a shallow tumor site and mitotic count ≥5/50 HPFs. However, whether this standard is applicable for discrimination between benign and malignant GGT needs to be further validated.

GGT is relatively rare. Previous reports are mostly dominated by one case, while the reports of a series of cases have gradually increased in recent years [26, 27]. It remains on debate whether the standard proposed by Folpe et al. [2] is applicable for GGT. To explore the prognostic significance of the clinicopathologic features, we statistically analyzed the clinicopathologic parameters of the total 31 cases, including the age, gender, maximum tumor diameter, involvement level, growth pattern, patchy consolidation, cellular atypia, atypical mitosis, vascular invasion, neural invasion, tumor necrosis, spindle cell area, and myxoid degeneration of the stroma. Univariate analysis revealed that patients with atypical mitosis and severe cellular atypia had shorter OS (*p* = 0.009, *p* = 0.007) and DFS (*p* = 0.010, *p* = 0.004), as compared to corresponding controls. Nevertheless, neither atypical mitosis nor severe cellular atypia exhibited prognostic significance for adverse clinical outcome of GGT patients in multivariate analysis. We believed that the prognostic significance of atypical mitosis and severe atypia for GGT is still worthy of further exploration, and the non-significant result might be associated with the low incidence of malignant GGT, requiring further large-scale studies. However, it is important to note that the assessment of nuclear atypia is subjective, and metastatic GGT has previously reported only mild cytologic atypia and a small amount of mitosis [3]. Therefore, Pansa et al. [26] believed that the absence of severe cell atypia and mitosis do not exclude malignant potential.

Some scholars pointed out that the stomach is a deep organ and over half the gastric tumors have a maximum diameter >2 cm (median, 2–3 cm) [3, 20]. Thus, they thought the standard mentioned above is not suitable for diagnosis of benign and malignant GGT. Miettinen et al. [3] believed that maximum tumor diameter >5 cm would increase the risk of developing recurrence/metastasis. Papke et al. [27] also recently conducted clinicopathological analysis on 26 cases of gastroesophageal glomus tumor, and proposed a new malignant criterion for gastroesophageal glomus tumor: 1) the maximum diameter of the tumor ≥5cm; or 2) both nuclear atypia and mitoses ≥2/10HPF. In the 15 cases reported here from Zhongshan hospital, the maximum tumor diameter was between 1–4 cm (average, 1.89 cm). One case had liver metastasis, while the maximum tumor diameter was 4 cm <5 cm. Of the 16 previously reported cases from different hospitals in the literature, the maximum tumor diameter ranged between 2–17 cm (median, 5.65 cm). While in the 9 cases developing metastasis, the maximum tumor diameter was between 3–17 cm (median, 6.5 cm; average, 7.23 cm). Besides, both the uni- and multi-variate analyses demonstrated that maximum tumor diameter ≥5 cm was not statistically associated with the adverse outcome of GGT. Perhaps more data may need to be accumulated to clarify the meaning. The prognostic significance of mitotic count was not covered in the current study, as the counting method for mitosis was not uniformed in the previously reported 16 cases from literature coverage (partial 10 HPFs, and partial 50 HPFs). The 16 cases of GGT reported in the previous literature did not mention the microscope model used, so only the data could be compiled for description. In all, the standard for differentiation between benign and malignant GT put forward by Folpe et al. [2] or Papke et al. [27] has certain limitations when applied for GGT cases.

In experience, vascular invasion, neural invasion, tumor necrosis, and presence of significant spindle cell area are potential indicators that predict the malignancy of tumor [28]. However, these appear to be less applicable in diagnosis of GGT cases. Haque et al. [29] held the view that the vascular invasion after GGT was not identical to the traditional vascular invasion with different clinical implications, and they believed that it was not applicable for predicting the malignancy of GGT. Among the 15 cases reported in the present study from Zhongshan hospital, 5 cases (20%) had vascular invasion, 6 cases (40%) experienced neural invasion, 1 case (7%) developed focal tumor necrosis, and 3 cases (20%) were detected with spindle cell areas in some tumor parts. While in the 16 previously reported cases of malignant GGT from literature coverage, 6 cases (38%) had partial spindle cell areas, 2 cases (15%) had partial tumor necrosis, and 6 cases (38%) developed vascular invasion. Our statistical analysis indicated that none of the events mentioned above had significant prognostic value for the adverse outcomes of GGT, but this can be also caused by the low number of involved cases. Of the 31 cases so far, 11 have vascular invasion, but the statistical results are not significant. We speculated that vascular invasion shown by GGT may only be a manifestation of local invasion of tumor tissue, but the potential to cause the planting and growth of corresponding organs is relatively limited. In addition, the 16 previously reported cases from literature review are dominated by single cases, resulting in incomplete morphological description. Besides, the fundamental cases of GGT and the incidence of patients developing adverse outcomes (recurrence/metastasis/death) within a certain period of time from the multi-center is not available, which leads to data deficiency or incompleteness. The conditions mentioned above increase certain difficulty of exploring the benign and malignant judgment criteria of GGT. In this context, the morphology of GGT is of certain clinical value that may potentially predict the adverse biological behaviors. Miettinen et al. [3] reported 32 gastrointestinal GT cases diagnosed from 1970 to 1998, including 31 GGT cases and 1 cecum GT case. Only one case had liver metastasis 33 months postoperatively and died 50 months postoperatively, histologically presenting with mild cellular atypia, spindle cell foci, vascular invasion, and 1 mitosis/50 HPFs. While in the 15 cases (2014–2022) reported here from Zhongshan hospital, 1 case had postoperative liver metastasis and histologically presented with vascular invasion, tumor necrosis, mitosis >5/50HPFs (12 mm^2^), and atypical mitosis. The above findings imply that GGT has a low incidence with few cases reported and has relatively indolent biological behaviors. Besides, more attention should be paid in cases displaying more high-risk indicators, which may predict the probability of developing adverse outcomes. In the meantime, close follow-up is also on demand to accumulate more data and experience to facilitate prediction of the biological behaviors of GGT.

Consistent with the present study, Luzar et al. [30] believed that the mitotic count at 50 HPFs was hard to be achieved in cutaneous GT due to the small tumor size in most cases, and that the standard proposed by Folpe et al. was not easily to be applied in cases of cutaneous GT. Similarly, Zhu et al. [31] reported a case of bronchial GT presenting with local infiltration, and mitotic count >5/50 HPFs (12-19 mitoses/50 HPFs, there was no evidence of recurrence or metastasis during the 2-year follow-up. This also indicated that the standard proposed by Folpe et al. is limited in predicting the biological behaviors of deep organs.

GGT shares similar IHC features with other GTs derived from soft tissues, majorly expressing α-SMA, vimentin, h-Caldesmon, calponin, and type IV collagen, partially expressing synaptophysin and CD34, and rarely expressing wide-spectrum cytokeratin, S-100 protein, CD117, desmin, CD56, and chromograninA, etc. [3, 32, 33]. Synaptophysin is mainly expressed in GGT but has not been reported in the GT of other sites, and there has been no literature reporting its prognostic significance [34]. Consistently, synaptophysin was expressed in 13 out of the 15 cases reported in the present study.

From the perspective of molecular genetics, previous study adopted fluorescent *in situ* hybridization (FISH) method and detected *NOTCH* gene rearrangement, dominated by *NOTCH2-MIR143* fusion gene (73%), in approximately 50% of GT cases [35]. Recent research reports that *CARMN-NOTCH2* fusion is also common (58%) [27]. Since fusion gene detection of *NOTCH* was not covered by our applied next-generation sequencing panel, there is no information in the present study. Another study found that approximately 6% malignant GT or atypical GT had *BRAF V600E* mutation, and *BRAF* gene was believed to be a potential therapeutic target [36]. To further clarify the genetic difference between benign and malignant GGT, the present study applied Sanger sequencing and ARMS testing but found no *BRAF V600E* mutation in either of the 15 cases. We reasoned that this might be due to the few cases of malignant GGT with liver metastasis (only 1 case here). Moreover, none of the 16 malignant GGT cases reported gene testing result, probably because the researchers were not aware of the prognostic significance of the *BRAF* gene in GT or due to the limited objective detection technique at that time. Next-generation sequencing reported *EGFR, PIK3CA, KEAP1* and *TP53* gene mutation*.* However, previous literature had not reported any prognostic significance of above gene mutations in GGT, so our study can only describe them.

To sum up, GGT is relatively indolent with good prognosis and usually managed by surgical resection in clinic. Notably, more attention should be paid to the presence of increasing morphological risk factors, which may predict the probability of developing adverse outcomes. In the meantime, long-term close follow-up is required to help timely find metastasis and thereby to increase the chance of prolonging survival time by surgical resection.

## Data Availability

The data supporting the findings of this study are available within the article and its supplementary material.
